# Synchronous mixed germ cell tumor of testis and renal cell carcinoma: A rare presentation

**DOI:** 10.1016/j.ijscr.2019.04.024

**Published:** 2019-04-16

**Authors:** Satish Deshmukh, Sushrut Fulare, Sanjeev Chowksey, Angir Soitkar, Akshay Nagre, Abhiram Mundle

**Affiliations:** NKP Salve Institute of Medical Sciences, Nagpur, India

**Keywords:** Renal cell carcinoma, Testicular tumor, Oncocytoma

## Abstract

**Introduction:**

Synchronous presentation of mixed germ cell tumor of testis and renal cell carcinoma is a rare presentation and has not been reported in literature. RCC accounts for 3% of all adult malignant neoplasms (Marzouk et al., 2014). It is the most lethal urologic cancers. Testicular cancer is of generally aggressive form and is the most common form of cancer in young men aged 15–35 years.

**Presentation of case:**

We report a case of incidental finding of left renal cell carcinoma with mixed germ cell tumor of right testis in our patient.

A 36 years old male patient came with complaints of painless swelling in the right side of the scrotum since 1 year. On palpation a hard swelling of size 5 x 4 cm was palpable in right scrotum. Ultrasonography suggested likelihood of a neoplastic etiology. On Contrast enhanced CT of abdomen, a well defined mass in the lower pole of left kidney likely to be an Oncocytoma, which was not palpable. CT guided biopsy of renal mass, was suggestive of epithelial renal tumor probably oncocytoma or low grade renal cell carcinoma.

**Discussion:**

In literature there are very few cases reported of metachronous mixed germ cell tumor of testis and renal cell carcinoma. There are 12 reports of patients having metachronous testicular and renal malignancy. During the metastatic work up to mixed germ cell tumor we found the mass in the left kidney which was ultimately diagnosed to be renal cell carcinoma. The management of both the malignancies depend on their merit and is a real challenge for a surgeon.

**Conclusion:**

Treatment strategies for both malignancies depend on accurate clinical staging and should be integrated to provide optimal results.

## Highlights

• Synchronous presentation of mixed germ cell tumor of testis and renal cell carcinoma is a rare presentation and has not been reported in literature.
• During the metastatic work up to mixed germ cell tumor we found the mass in the left kidney which was diagnosed to be renal cell carcinoma.
• Treatment strategies for both malignancies depend on accurate clinical staging and should be integrated to provide best results.
• The management of both the malignancies depend on their merit and is a real challenge for a surgeon.

## 1 Introduction

Testicular tumors are a heterogeneous group of neoplasms exhibiting diverse histopathology, variable clinical course and prognosis. Of these tumors, 30–50% are classified as mixed germ cell tumors. Renal cell carcinoma (RCC) is a lethal tumour that accounts for about 3% of all adult malignancies [1]. This is primarily a disease of the elderly patient, with typical presentation in the sixth and seventh decades of life [2]. About 25%–30% of patients will present with metastatic disease at the time of diagnosis. Testicular metastasis from RCC is extremely rare. Because of the paucity of literature on the topic, little is known with regards to the patterns of spread and the asso- ciation between metastatic RCC and dissemination to the testes [1]. Synchronous occurrence of renal cell carcinoma and mixed germ cell tumor of testis has not been reported in literature. We report a case of incidental finding of renal cell carcinoma in a diagnosed case of mixed germ cell tumor of testis.

The work has been reported in line with the SCARE criteria [3].

## 2 Case report

A 36 years old male patient came with complaints of swelling in the right side of the scrotum since 1 year not associated with pain. The right side scrotal swelling was gradually progressive and on palpation it was hard and non tender. USG of the scrotum was done, which showed a well defined heterogenous legion of size 6.4 × 5.9 × 4 cms in the right scrotal sac arising from the lower pole of right testis with multiple cystic areas within suggestive of likely neoplastic etiology. Blood tumor markers showed raised levels- B-HCG 27.76 miu/ml, A.F.P- 251.69 ng/ml, Sr. LDH-642 units/L.

A Contrast enhanced CT of the abdomen showed a well defined heterogenous lesion of size 4.8 × 3.4 cms is noted arising from the lower pole of left kidney of a possible neoplastic etiology.

**Fig. 1:**
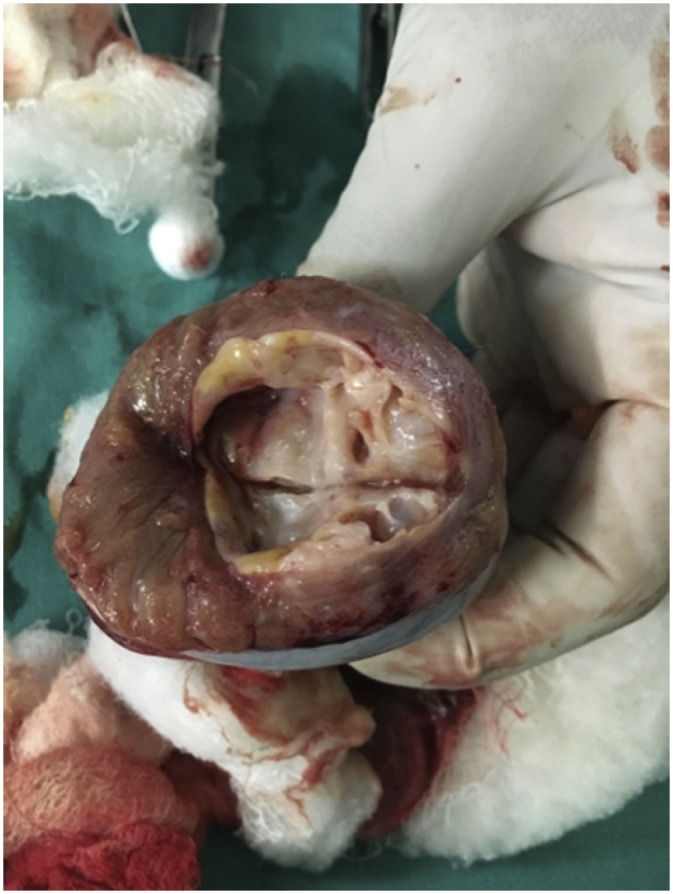
Post operative picture after orchidectomy.

Patient underwent right sided high inguinal orchidectomy and the specimen was sent for histopathological examination (Fig. 1). On gross and microscopic examination, it was revealed that the specimen is suggestive of mixed germ cell tumor: embryonal carcinoma, teratoma and seminoma.

Later, a CT guided core biopsy of the left renal mass was done. The biopsy report suggested epithelial renal tumor, probably oncocytoma/low grade renal cell carcinoma (RCC).

Patient was given six chemotherapy cycles of Etoposide-Cisplatin regimen. After completing Etoposide-Cisplatin regimen patient underwent a review CT abdomen which suggested of a heterogenous mass from lower pole of left kidney like Oncocytoma? ? Renal cell carcinoma.

**Fig. 2:**
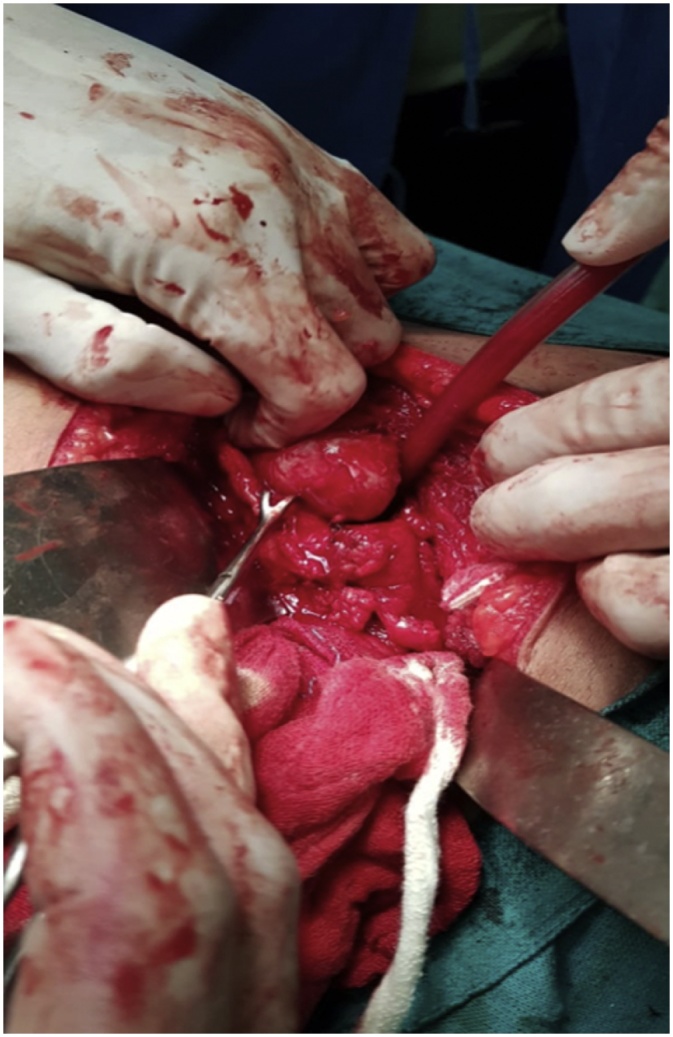
Patient undergoing partial nephrectomy.

The patient underwent left partial Nephrectomy(Fig. 2). The histopathological examination of which was suggestive of clear cell variant of renal cell carcinoma – Grade II.

## 3 Discussion

Renal cell carcinoma, the most common type of renal malignant disease, accounts for 2%–3% of all adult malignant neoplasms [4]. The majority of renal malignant neoplasms are now diagnosed incidentally by cross-sectional imaging or ultrasound evaluation of other nonspecific complaints [5]. Historically, renal cell carcinoma was diagnosed only at an advanced stage because of its location within the retroperitoneum. Despite this increase in asymptomatic diagnoses, 30% of patients present with metastatic disease [6].

**Table:** 

1^st^ Author	Primary testis tumor	Renal tumor	Radiotherapy	Time RCC occurred after primary
Lawrentschuk [7]	Mixed GCT	RCC	No	1.5years
Travis [8]	10 Seminomas	RCC	Yes- 6 cases	NA
			No- 4 cases	
Dieckmann [9]	1 Seminoma	RCC	Yes	6 years
Davis [4]	1 Seminoma	RCC	Yes	3 years
Van Leeuwen [10]	4 Seminomas	RCC	NA	NA
Moller [11]	16 Seminomas	RCC	NA	8 at 0-9 years
				4 at 10-19 years
				others NA
Deshmukh(This study)	1 Mixed GCT	RCC	NA	Synchronous

The finding of a new lesion in a kidney, in the setting of a known testicular tumour, raises the possibility of metastatic disease. Testicular tumours rarely present clinical evidence of spread to the kidney, although autopsy studies have found up to 25% of non seminomatous tumours to have progressed to renal metastasis, thus this condition needs to be excluded. There are 12 reports of patients having simultaneous testicular and renal malignancy [4,9,12,13]. Metachronous malignant neoplasms developing after treatment for testicular germ cell tumours are uncommon, but the development of a renal cell carcinoma after a previous testicular tumour is particularly rare. An increased incidence of renal cancer in long term survivors of testicular cancer has been suggested [14]. Harris and Suemasu proposed that multiple neoplastic disease arises in patients due to an oncogenetic susceptibility, probably due to genetic factors [15]. Possible contributing factors to this risk of second malignancy may also include radiation-induced solid tumours and chemotherapy-induced leukaemia.

## 4 Conclusion

Although this case represents a rare entity of a simultaneous primary renal and testicular malignancy, an individual patient not only can present a diagnostic dilemma but also can raise questions regarding appropriate management. With advancements in molecular biology and the mapping of the human genome, analysing the genotype of patients with second primary malignancies should provide further insight into the genetic aetiology of such tumours or whether they occur purely by chance. Treatment strategies for both malignancies depend on accurate clinical staging and should be integrated to provide optimal results.

## Conflicts of interest

None.

## Funding

None.

## Ethical approval

Yes, NKP Salve Institute of Medical Sciences.

IEC/NKP SIMS-2/2018.

## Consent

Written informed consent was obtained from the patient for publication of this case report and accompanying images. A copy of the written consent is available for review by the Editor-in-Chief of this journal on request.

## Author contribution

Main operating surgeon: Dr. S.D. Deshmukh.

Assitant operating surgeon: Dr. Sushrut Fulare.

Second assisting surgeon:Dr. Sanjeev Chowksey.

Data collection: Dr. Abhiram Mundle, Dr Angir Soitkar.

Writing: Dr. Abhiram Mundle, Dr. Akshay Nagre.

## Registration of research studies

As this was not a human study, the registration of research studies was not obtained.

## Guarantor

Dr. Satish Deshmukh.

## Provenance and peer review

Not commissioned, externally peer reviewed.
